# Unveiling the role of localized polaronic mid-gap states in enhanced carrier transfer in TiO_2_/BiVO_4_ heterojunctions under visible light irradiation

**DOI:** 10.1038/s41598-025-10259-9

**Published:** 2025-07-08

**Authors:** Zixi Yin, Xingchen Liu, Guijie Liang, Yin Wang

**Affiliations:** 1 Hubei Key Laboratory of Low Dimensional Optoelectronic Materials and Devices, Hubei University of Arts and Science, Xiangyang, 441053 Hubei China; 2 Institute of functional materials, Hubei University of Arts and Science, Xiangyang, 441053 Hubei China; 3 Hubei Longzhong Laboratory, Xiangyang, 441053 Hubei China; 4 School of Physics and Electronic Engineering, Hubei University of Arts and Science, Xiangyang, 441053 Hubei China

**Keywords:** Mid-gap states, Heterojunction, Interfacial carrier transfer, Photocatalytic mechanism, Chemistry, Physics

## Abstract

TiO_2_/BiVO_4_ heterojunctions are considered to be one of the most promising materials for photocatalysts due to their extended carrier lifetime, high visible light response, and good stability. However, while Type-II TiO_2_/BiVO_4_ heterojunctions are well-studied, the fundamental mechanism behind the Type-I configurations remains unclear, particularly regarding their unexpected high photocatalytic activity despite theoretically unfavorable band alignment. Herein, we reveal that localized polaronic mid-gap states (SP states) can mediate efficient charge transfer and recombination in TiO_2_/BiVO_4_ using time-resolved photoluminescence (PL) spectroscopy and transient absorption spectroscopy (TAS), providing direct experimental evidence of this mechanism. The existence of SP states enables exceptional methyl orange degradation efficiency (nearly 100% in 1 h under visible light) despite the theoretically unfavorable Type-I alignment. This work redefines the potential of Type-I systems for visible-light photocatalysis by demonstrating how polaron engineering overcomes the limitations of traditional band structures, advancing their applications in solar utilization.

**Supplementary Information:**

The online version contains supplementary material available at 10.1038/s41598-025-10259-9.

## Introduction

Photocatalytic processes are one of the most attractive ways to utilize solar energy, which is of great significance to solve the current energy crisis and environmental pollution. Constructing composites with heterojunction structure has proved to be an effective means to increase the utilization of sunlight and prolong the carrier lifetime of photocatalysts, which ultimately significantly improves the photocatalyst efficiency^1–4^. Several high-performance heterojunction photocatalysts have been reported, exemplified by WO_3_/BiVO_4_^5^, BiOBr/CuInS_2_/WO_3_^6^ and Bi_2_MoO_6_/g-C_3_N_4_/Ag_2_MoO_4_^7^, which demonstrate enhanced photocatalytic performance through optimized charge separation. Among various heterojunctions, TiO_2_/BiVO_4_ has emerged as a particularly promising system by synergistically addressing the inherent limitations of its components: while TiO_2_ suffers from poor visible-light absorption and rapid carrier recombination^8^, recent advances in defect engineering (e.g., oxygen vacancy-modulated TiO_2_) have enhanced its photoresponse^9^; Meanwhile, the short carrier lifetime and diffusion length of BiVO_4_ have been improved through nanostructuring and doping strategies (e.g., Al-doped BiVO_4_ achieves nearly 3× longer diffusion length and carrier lifetime)^10^. The TiO_2_/BiVO_4_ heterojunctions combine TiO_2_’s ideal conduction band edge, exceptional chemical stability, and biocompatibility with BiVO_4_’s visible-light harvesting (E_g_ ≈ 2.4 eV), overcoming shortcomings of both materials through interfacial charge engineering^11–14^.

**Fig. 1:**
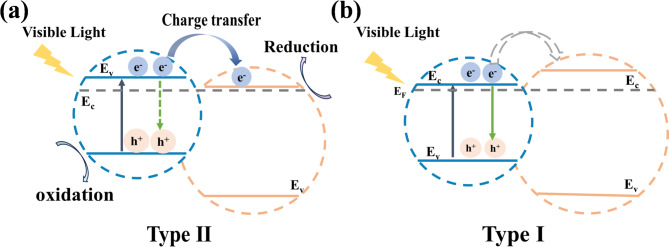
The energy barrier at the interface is unfavorable to carrier transfer. Schematic diagram of carrier transfer in heterojunctions with (**a**) Type II and (**b**) Type I energy level structures under visible light irradiation.

Interfacial charge transfer has been proved to critically determine the photocatalytic performance of TiO_2_/BiVO_4_ heterojunctions. In conventional Type-II configurations (Fig. 1a), the staggered band alignment drives electrons to migrate from BiVO_4_ to TiO_2_ while holes remain in BiVO_4_, resulting in spatial separation of photogenerated electrons and holes for efficient redox reactions^13^. For example, these separated carriers can directly participate in pollutant degradation: holes on the valence band (VB) of BiVO_4_ can oxidize organic pollutants (e.g., MO and MB) through •OH radical formation or directly decompose them via hole-transfer pathways, while electrons on the conduction band (CB) of TiO_2_ can reduce O_2_ to O_2_^•−^ for further oxidative chain^15– 16^. Specifically, Feng and Fu et al. observed long-lived charge-separated states in Type-I TiO_2_/BiVO_4_, where photoexcited high-energy electrons from BiVO_4_ can possess sufficient energy to overcome the narrow potential barrier between TiO_2_^17^. Despite significant advances in TiO_2_/BiVO_4_ heterojunction design, the charge dynamics under visible-light excitation, particularly how carriers overcome the large interfacial energy barriers in Type-I heterojunctions (Fig. 1b) and the influence of TiO_2_’s mid-gap states, remain poorly understood. Addressing this gap is fundamentally important, as mechanistic insights into TiO_2_/BiVO_4_’s charge transfer could directly guide interface engineering to enhance both visible-light absorption and carrier separation efficiency, which are the dual requirements of high-performance photocatalysis.

**Fig. 2:**
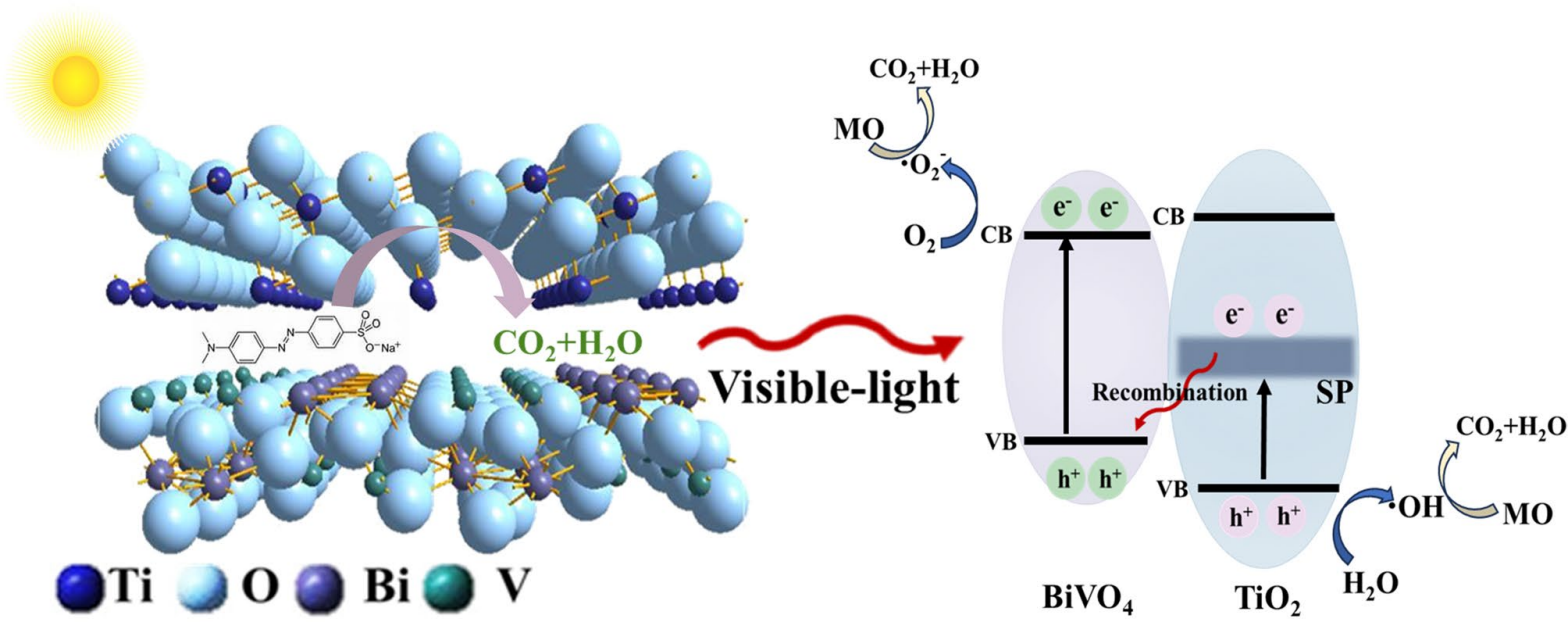
Graphical abstract: Enabling superior visible-light photocatalytic degradation of methyl orange in ‘inefficient’ Type-I TiO_2_/BiVO_4_ heterojunction via polaron engineering.

In this work, we synthesized a TiO_2_/BiVO_4_ heterojunction with Type-I band structure and explored the mechanisms of carrier transfer, recombination and photocatalysis under visible light irradiation with phonon energy lower than the band gap of TiO_2_. Efficient charge migration pathway mediated by the localized polaronic mid-gap states of TiO_2_ was confirmed by structural and time-resolved spectroscopy results, which can effectively separate the photogenerated carriers of BiVO_4_ and TiO_2_, and eventually lead to the substantial improvement on the visible photocatalytic performance of TiO_2_/BiVO_4_ relative to pure BiVO_4_ and TiO_2_ (Fig. 2). Long-lived photogenerated holes in TiO_2_ and electrons in BiVO_4_ were both observed, and the degradation rate of methyl orange (MO) reached nearly 100% under weak visible light irradiation. This work broadens the feasible way to synthesize TiO_2_/BiVO_4_ photocatalysts for efficient use of solar energy and provides a new idea for improving the visible light utilization rate of TiO_2_.

## Methods

All of the materials used in the experiments were purchased from Inno-chem and used without further purification.

### Synthesis of BiVO_4_

The BiVO_4_ nanocatalysts were synthesized using a simple coupling method. In a typical procedure, 0.02 mol Bi(NO_3_)_3_·5H_2_O was dissolved in 30 mL HNO_3_ (4 mol/L). The mixture was denoted as solution A. 0.01 mol of NH_4_VO_3_ was dissolved in 30 mL of NaOH solution (1 mol/L) (solution B). Subsequently, solution A was then added dropwise to solution B with the addition of 1 g ethyl cellulose. Followed by the pH adjustment with an NaOH solution (2 mol/L), until the mixture reached a pH of 6. After stirring for 1 h, the mixture was transferred into a Teflon-lined stainless steel autoclave with a capacity of 100 mL. Then, the sealed reactor was heated to 180 ^◦^C for 8 h. The final products were filtered and washed with deionized water and ethanol several times, and finally dried at 100 °C for 8 h.

### Synthesis of TiO_2_

A mixture of 2 mL HNO_3_ and 6 mL ethyl alcohol was added dropwise to the mixture of 20 mL butyl titanate and 30 mL ethyl alcohol with sustained stirring for 12 h to form a highly dispersed colloidal solution. Subsequently, the colloid was dried overnight in the oven and then was calcined at 500 °C for 2 h.

### Synthesis of TiO_2_/BiVO_4_

The procedure for the synthesis of the nanocomposites was conducted according to the BiVO_4_ synthesis protocol. A certain amount of as-prepared TiO_2_ was added into the precursor solution of bismuth vanadate prior to the pH adjustment. The molar ratio of Ti and Bi was fixed to 1 : 0.06.

### Femtosecond transient absorption measurement

The femtosecond transient absorption setup used for this study is based on a regenerative amplified Ti: sapphire laser system from Coherent (800 nm, 35 fs, 6 mJ pulse^− 1^, and 1 kHz repetition rate), nonlinear frequency mixing techniques and the Femto-TA100 spectrometer (Time-Tech Spectra). Briefly, the 800 nm output pulse from the regenerative amplifier was split into two parts with a 50% beam splitter. The transmitted part was used to pump a TOPAS Optical Parametric Amplifier (OPA) which generates a wavelength tunable laser pulse from 250 nm to 2.5 μm as a pump beam. The reflected 800 nm beam was split again into two parts. One part with less than 10% was attenuated with a neutral density filter and focused into a 2 mm thick sapphire window to generate a white light continuum (WLC) from 420 nm to 800 nm used for the probe beam. The probe beam was focused with an Al parabolic reflector onto the sample. After the sample, the probe beam was collimated and then focused into a fiber-coupled spectrometer with CMOS sensors and detected at a frequency of 1 kHz. The delay between the pump and probe pulses was controlled by a motorized delay stage. The pump pulses were chopped by a synchronized chopper at 500 Hz and the absorbance change was calculated with two adjacent probe pulses (pump-blocked and pump-unblocked). All experiments were performed at room temperature.

### PL spectra and steady-state absorption measurements

PL spectra were obtained by using F-4700 Hitachi fluorescence spectrophotometer. Time-resolved PL spectra were recorded by FLS980 multifunction steady-state and transient-state fluorescence spectrometer. The UV-vis absorption spectra were recorded using a UV-Vis spectrometer (UV-3700, Shimadzu, Japan) with BaSO_4_ as a reference.

### Structure characterization

The X-ray diffraction (XRD) patterns were obtained on the D8 Advance X-ray diffractometer and equipped with a Cu-Kα radiation. The data were expressed in the 2θ range from 20 ° to 80 °.

X-ray photoelectron spectra (XPS) were acquired on the Thermo Scientific escalab 250xi system with Al Kα (1486.6 eV) as the excitation source.

The morphology of the samples was explored using a Hitachi S4800 scanning electron microscope (SEM) (accelerating voltage 5 kV). The Energy Dispersive Spectra (EDS) were measured on HORIBA EX-250 instrument.

TEM images were obtained on a Talos F200X transmission electron microscope.

### Electrochemical measurements

Electrochemical impedance and photocurrent measurements were conducted with a CHI660e electrochemical station in a typical three electrode cell, using a Pt sheet as the counter electrode and a saturated Ag/AgCl as the reference electrode. The working electrode was prepared by spreading the ethanolic slurry of photocatalyst over ITO plate with an area of 3.5 cm^2^. The suspension was prepared by dispersing the photocatalyst (20 mg) and ethyl cellulose (2 mg) into a mixed solution containing 1 mL ethanol. A 300 W Xe lamp with a λ > 420 nm optical filter was utilized as the light source and the electrolyte was an aqueous solution of Na_2_SO_4_ (0.5 M).

### Photocatalytic test

The photocatalysis experiment was processed in multichannel photochemical reaction system (CEL-LB70, Ceaulight) with 300 mW/cm^2^ Xe lamp light source. The photocatalysis of visible light was carried out with a λ > 420 nm optical filter. The photocatalytic activity was studied by preparing 20 mg/L methyl orange (MO) as the experimental mode. 0.1 g of sample was added to 100 mL MO solution with continuous stirring. Before the photocatalytic experiment, the reaction system was treated in the dark for 30 min to achieve the adsorption-desorption equilibrium. After the light reaction for a certain time, 5 mL suspension was taken and separated by centrifugation. The concentration of MO was determined by spectrometer.

## Results and discussion

### Characterization

**Fig. 3:**
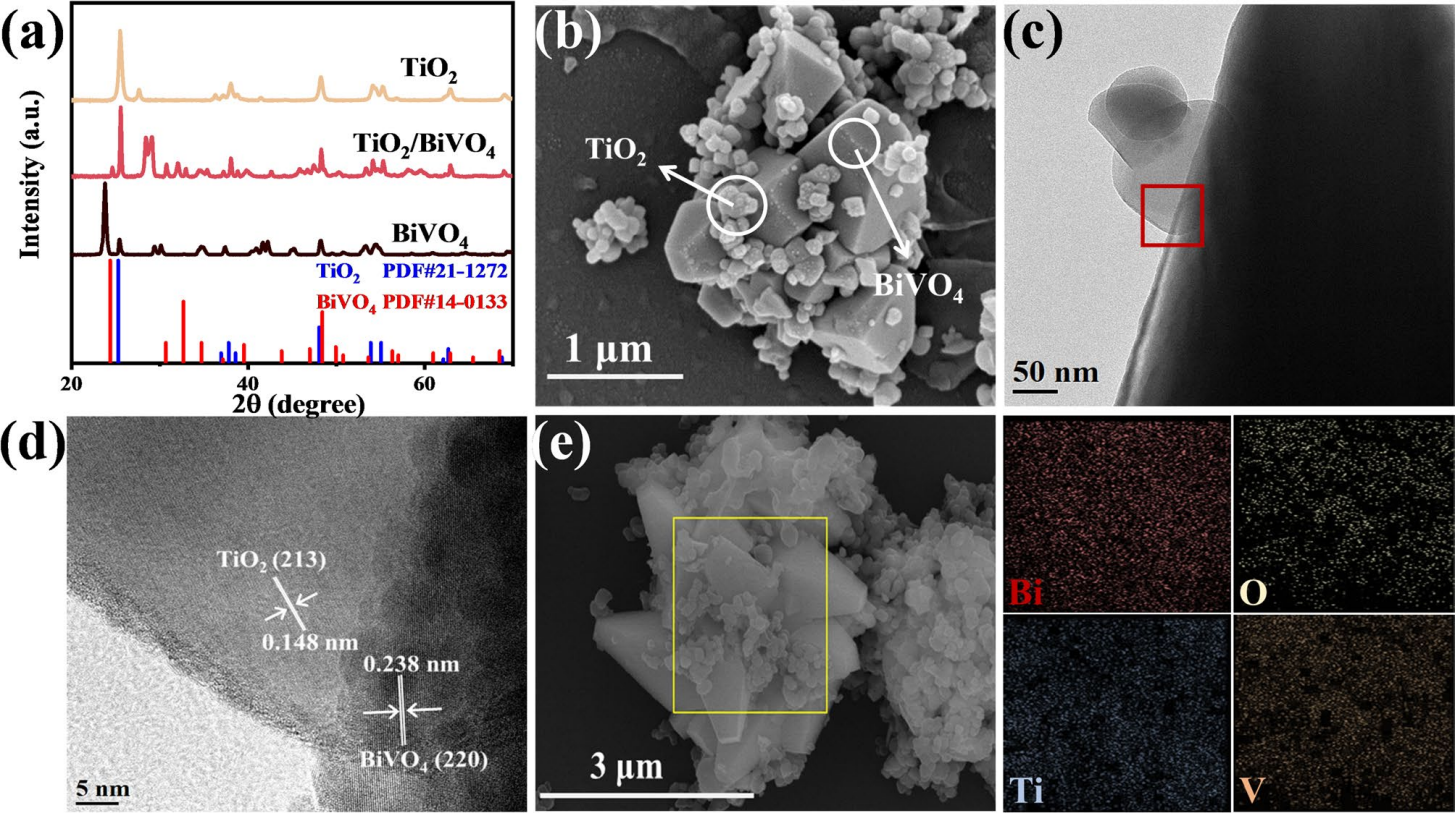
The TiO_2_/BiVO_4_ composite heterojunction was successfully synthesized. (**a**) XRD patterns of BiVO_4_, TiO_2_ and TiO_2_/BiVO_4_. (**b**) SEM image of TiO_2_/BiVO_4_, the spherical TiO_2_ particles are deposited on the BiVO_4_ polyhedron, indicating the successful synthesis of the TiO_2_/BiVO_4_ composite. (**c**) TEM and (**d**) High-resolution TEM (HRTEM) image of the selected area (the red square in Fig. 3c) of a typical TiO_2_/BiVO_4_. (**e**) The elemental mapping diagrams of TiO_2_/BiVO_4_.

Herein, nanocomposites were synthesized using modified methods as previously reported (see Methods for the details)^18,19^. Powder X-ray diffraction (XRD) was employed to study the phase and crystallographic features of synthesized samples. The XRD diffraction patterns for TiO_2_/BiVO_4_, pure TiO_2_ and BiVO_4_ are shown and compared in Fig. 3a. The diffraction peaks of the BiVO_4_ powder are in great agreement with those of the monoclinic phase, while the diffraction peaks of the prepared TiO_2_ corresponding to an anatase are clearly observed. The TiO_2_/BiVO_4_ exhibits both the characteristic peaks of BiVO_4_ and TiO_2_, indicating the successful preparation of TiO_2_/BiVO_4_. The diffraction peaks at 2θ values of 25.56 ^o^, 48.26 ^o^, 54.08 ^o^ and 62.87 ^o^ of the TiO_2_/BiVO_4_ correspond to the diffraction related to the anatase structure, with the planes (101), (200), (105) and (213). Meanwhile, the 2θ values of 24.63 ^o^, 30.74 ^o^, 32.93 ^o^, 34.54 ^o^ and 48.28 ^o^ can be attributed to diffraction related to the planes (200), (211), (112), (220) and (312) of BiVO_4_. In addition, the diffraction peaks with high intensity and narrow linewidth indicate that the prepared samples have good crystalline features.

The microstructures and microscopic morphologies of the as-prepared crystals were characterized by scanning electron microscopy (SEM) and transmission electron microscopy (TEM). As confirmed by SEM images shown in Fig. 3b and Fig. S1, the irregular TiO_2_-spherical particles (Fig. 3b and S1a) are attached to the BiVO_4_-polyhedron (Fig. 3b and Fig. S1b) to form TiO_2_/BiVO_4_ compounds. The TEM image of the TiO_2_/BiVO_4_ composite shown in Fig. 3c provides the similar morphological feature as previous SEM images. Two different kinds of lattice fringes and the clear contact interface can be observed on the area selected high-resolution TEM (HRTEM) image exhibited in Fig. 3d, the facet distances of 0.148 nm and 0.238 nm correspond to the (213) crystallographic plane of TiO_2_ and the (220) lattice plane of BiVO_4_, respectively, which confirms the intimate interfacial contact of TiO_2_ and BiVO_4_ and the formation of heterojunction structure in TiO_2_/BiVO_4_. Furthermore, the elemental mapping of TiO_2_/BiVO_4_ heterostructures also suggests that all the elements (Bi, O, V and Ti) are uniformly distributed over the entire composite (Fig. 3e), confirming the successful synthesis of TiO_2_/BiVO_4_ again.

**Fig. 4:**
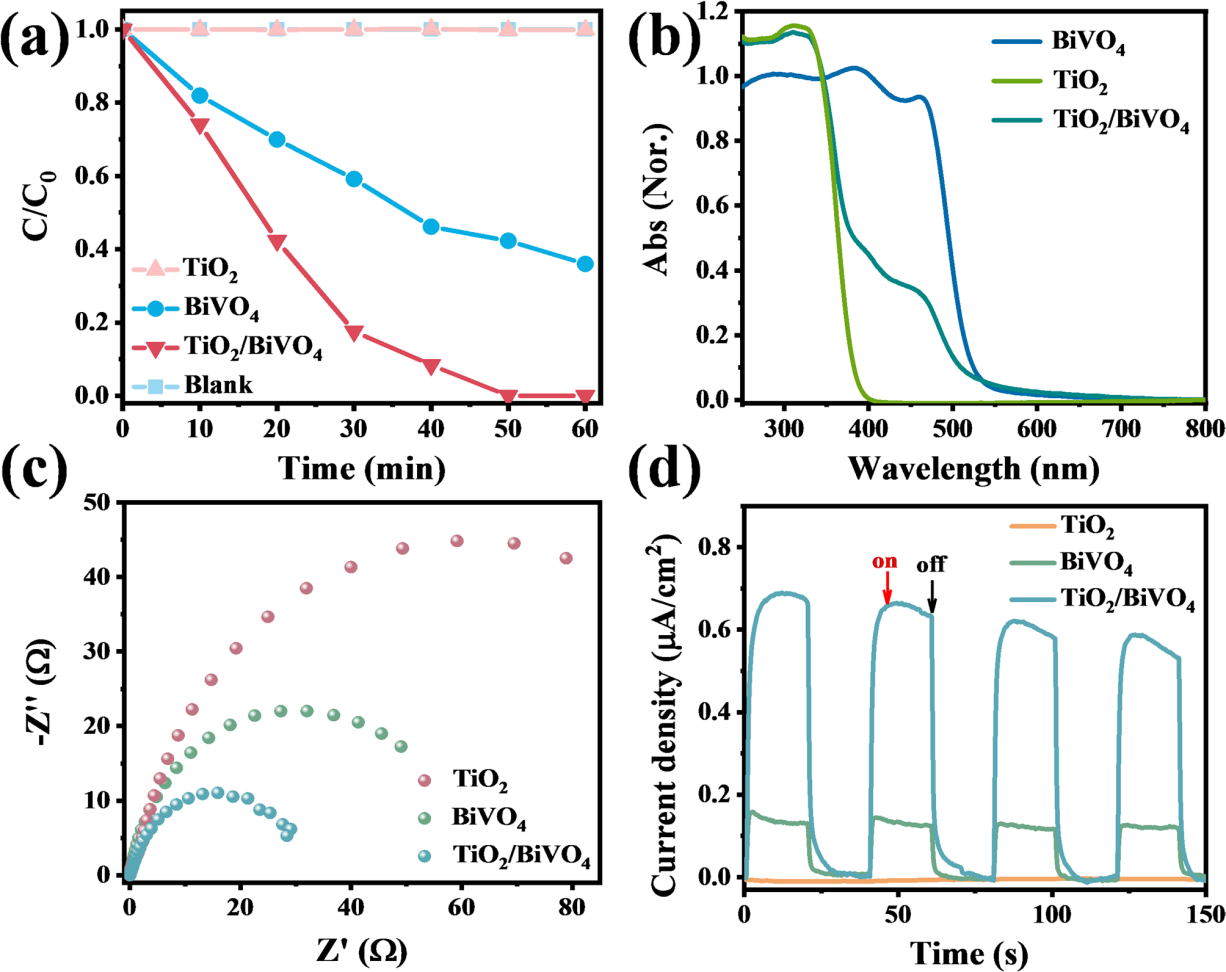
(**a**) Photocatalytic degradation of MO over pure TiO_2_, BiVO_4_ and TiO_2_/BiVO_4_. (**b**) Comparison of steady-state UV-vis absorption of TiO_2_, BiVO_4_ and TiO_2_/BiVO_4_. (**c**) Electrochemical impedance spectroscopy (EIS) Nyquist plots and (**d**) Transient photocurrent response under visible light irradiation of TiO_2_, BiVO_4_ and TiO_2_/BiVO_4_.

We then evaluated the photocatalytic performances of the BiVO_4_, TiO_2_ and TiO_2_/BiVO_4_ in terms of the photocatalytic degradation of methyl orange (MO) under visible light irradiation. Figure 4a shows the evolution of methyl orange (MO) concentration over time in the presence of different photocatalysts. Pure TiO_2_ exhibits almost no photocatalytic activity for methyl orange (MO) degradation under visible light irradiation, while the photocatalytic MO degradation rate of pure BiVO_4_ is ~ 64%. Besides, the photocatalytic activity of TiO_2_/BiVO_4_ composite is significantly improved and the photocatalytic degradation rate of methyl orange reaches nearly 100% under the same condition. The UV–vis absorption spectra of BiVO_4_, TiO_2_ and TiO_2_/BiVO_4_ under the same conditions are firstly compared in Fig. 4b to judge the influence of light absorption capacity on the improvement of photocatalytic performance. The TiO_2_/BiVO_4_ exhibits a decrease of photon absorption relative to pure BiVO_4_ in the visible light region, which is contrary to the results of photocatalysis, indicating that the improvement in photocatalysis performance is not due to the difference in light absorption capacity.

Meanwhile, electrochemical impedance spectroscopy (EIS) and photocurrent measurements were also performed to evaluate the charge transfer capabilities of the composites. As depicted in Fig. 4c, TiO_2_/BiVO_4_ exhibits the smallest semicircle capacitance compared to pure TiO_2_ and BiVO_4_, which implies a reduced charge-transfer resistance and the fastest interfacial charge transfer. The I-t curve of TiO_2_/BiVO_4_ also shows the largest photocurrent for four successive light-on and off cycles, while pure TiO_2_ has almost no response under the same condition, indicating the highest carrier separation efficiency of BiVO_4_ in TiO_2_/BiVO_4_ compared to the single component (Fig. 4d). The above results lead us to speculate that the more efficient charge separation and transfer should be the major mechanism for the enhanced photocatalytic performance of TiO_2_/BiVO_4_.

### The determination of localized polaronic mid-gap states

**Fig. 5:**
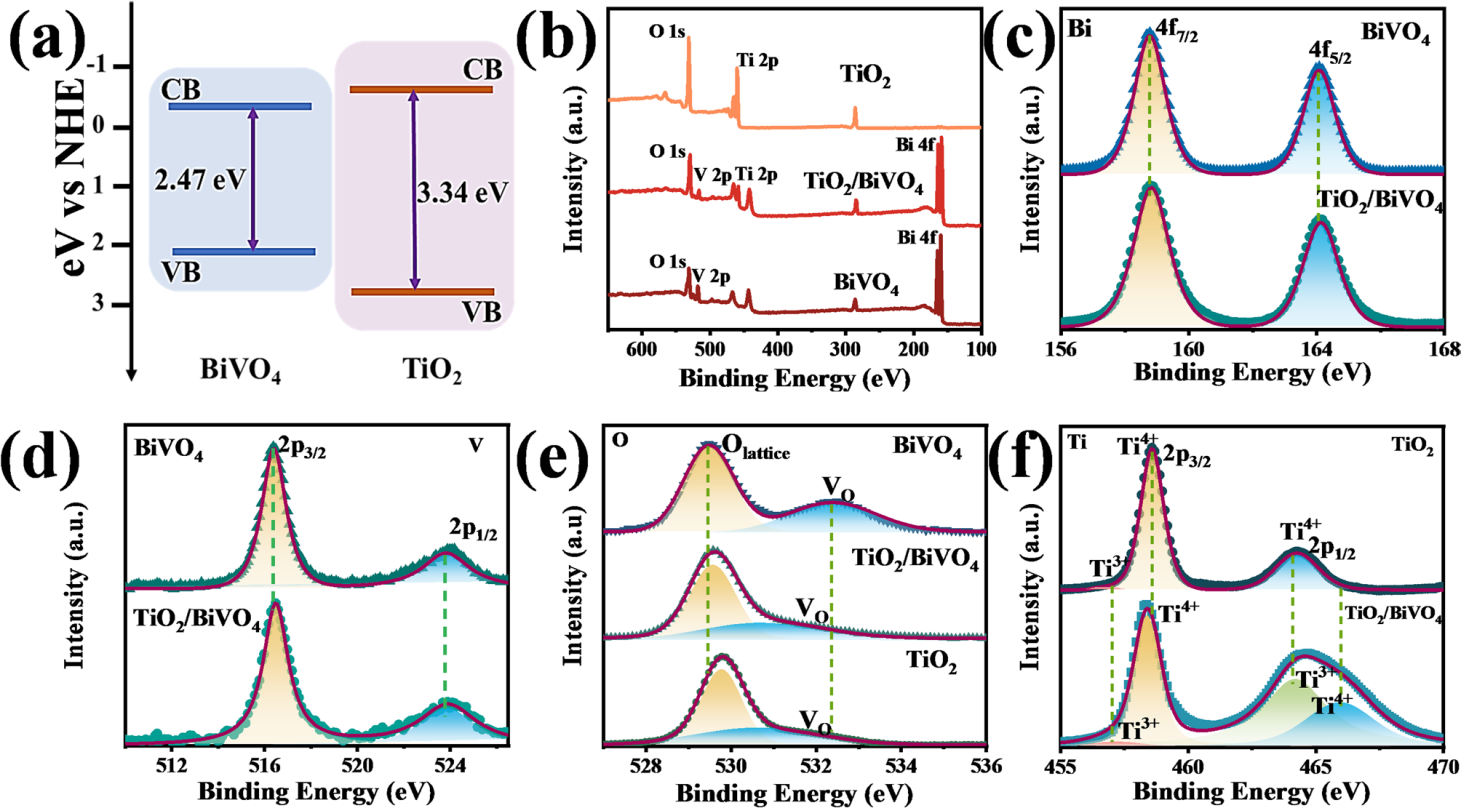
The synthesized TiO_2_/BiVO_4_ nanocomposites exhibit a type I band alignment and increased O-vacancy density. (**a**) The energy diagram of TiO_2_/BiVO_4_. (**b**) The survey spectra of pure TiO_2_, BiVO_4_ and TiO_2_/BiVO_4_. The high-resolution XPS profiles of (**c**) Bi 4f, (**d**) V 2p, (**e**) O 1s and (**f**) Ti 2p, in TiO_2_, BiVO_4_ and TiO_2_/BiVO_4_.

The band structure closely related to the charge transfer process is shown in Fig. 5a, the synthesized TiO_2_/BiVO_4_ nanocomposites exhibit a type I band alignment, where both the electron (ET) and hole (HT) transfer from BiVO_4_ to TiO_2_ are indeed unfavorable due to the energy barrier that exists at the interface. According to the Tauc plots (Figure S2a) and the Mott-Schottky tests (Figure S2b), the optical band gaps (E_g_) were determined to be 3.34 eV for TiO_2_ and 2.47 eV for BiVO_4_, while the conduction bands (CB) are estimated to be -0.69 eV for TiO_2_ and − 0.61 eV for BiVO_4_ (vs. NHE) based on Eq. (1).

1 $$E_{{NHE}} = E_{{Ag/AgCl}} + {\text{ }}0.{\text{197}}$$

In this energy level arrangement, there should be almost no charge transfer between BiVO_4_ and TiO_2_ under our experimental conditions, since the white light source we used (λ ≥ 420 nm, E_photon_ ≤ 2.95 eV) can only excite electrons in BiVO_4_ to the CB, but not TiO_2_, which is inconsistent with the phenomena we observed in photocatalysis, ESI and photocurrent experiments. Hence, it is reasonable to assume that there might be another carrier transport and recombination mechanism in TiO_2_/BiVO_4_.

Previous reports suggest that charge carriers produced by surface traps (O vacancies, etc.) can introduce localized polaronic mid-gap states in TiO_2_, i.e., excess electrons in the Ti sublattice near the O vacancy produce reduced Ti^3+^ centers, thus facilitating the formation of small polaron^20–24^. These localized small-polaronic (SP) states are estimated to be more than one eV above the VB edge of TiO_2_, which can not only enhance the visible light absorption of TiO_2_ but also induce different carrier traveling, trapping and recombination pathways. Moreover, the presence and increased density of O-vacancy in the synthesized TiO_2_/BiVO_4_, as well as the interaction between TiO_2_ and BiVO_4_, were affirmed by the XPS analysis. Thus, we suspected that it might be necessary to consider the influence of O-vacancy induced localized SP states.

The survey spectra of pure TiO_2_ and BiVO_4_ exhibit Ti, O and Bi, V, O elements, respectively, while TiO_2_/BiVO_4_ contains the constituent elements of both TiO_2_ and BiVO_4_ (Fig. 5b). The high-resolution Bi 4f spectra exhibit two feature peaks around ~ 164.1 eV (Bi 4f_5/2_) and ~ 158.8 eV (Bi 4f_7/2_) in both BiVO_4_ and TiO_2_/BiVO_4_ (Fig. 5c), indicating that Bi^3+^ is the main bismuth species in both samples^25^. In Fig. 5d, two peaks centered at the binding energy of ~ 516.4 eV (V 2p_3/2_) and ~ 523.8 eV (V 2p_1/2_) show a slight blue shift in TiO_2_/BiVO_4_ compared with BiVO_4_, demonstrating the existence of the characteristic V^5+^ in both compounds and the interfacial interactions between TiO_2_ and BiVO_4_ in TiO_2_/BiVO_4_ composite^4^. Furthermore, the O 1s spectra of both pure BiVO_4_ and TiO_2_ can be deconvolved into two separate peaks (Fig. 5e), where peaks at low binding energy (529.5 eV, 529.8 eV) can be assigned to the O^2−^ species in the lattice (O_lattice_) and the high binding energy peaks (530.6 eV, 532.4 eV) can be corresponded to hydroxyl groups bound to metal cations in the oxygen-deficient region (O_v_)^26^. In the case of TiO_2_/BiVO_4_, the binding energy of O_lattice_ is ~ 529.6 eV, which is located between BiVO_4_ and TiO_2_ and suggests the change of lattice environment. Meanwhile, the binding energy of O_v_ is close to pure TiO_2_, along with a slight increase in intensity, illustrating that the denser O-vacancy mainly exists in the TiO_2_ component for TiO_2_/BiVO_4_ composite.

The effect of O-vacancy can also be reflected by the XPS spectra of Ti 3d, as the creation of Ti^3+^ centers is closely related to the O-vacancies^27^. As shown in Fig. 5f, two peaks at 458.6 eV and 464.3 eV are observed for pure TiO_2_, which represent Ti 2p_3/2_ and 2p_1/2_ for Ti^4+^ in TiO_2_, respectively^28^. Along with these two peaks, a weak shoulder peak attributed to the Ti^3+^ center emerges at 456.8 eV^29^. All deconvoluted Ti 2p spectra show a shift of binding energy in TiO_2_/BiVO_4_, validating the formation of heterojunction structure. Notably, the shoulder peak assigned to Ti^3+^ 2p_3/2_ (~ 457.2 eV) becomes more intense and another new distinct peak belonging to Ti^3+^ 2p_1/2_ appears at 464.2 eV after combining with BiVO_4_ (Fig. 5f, lower panel), indicating the formation of Ti^3+^ is of greater preference in TiO_2_/BiVO_4_ and is consistent with the apparent O_v_ peak shown in the O 1s XPS spectra of TiO_2_/BiVO_4_, which further confirms the existence and higher density of O-vacancy in TiO_2_/BiVO_4_, and suggests the possible existence of small-polaronic mid-gap states.

**Fig. 6:**
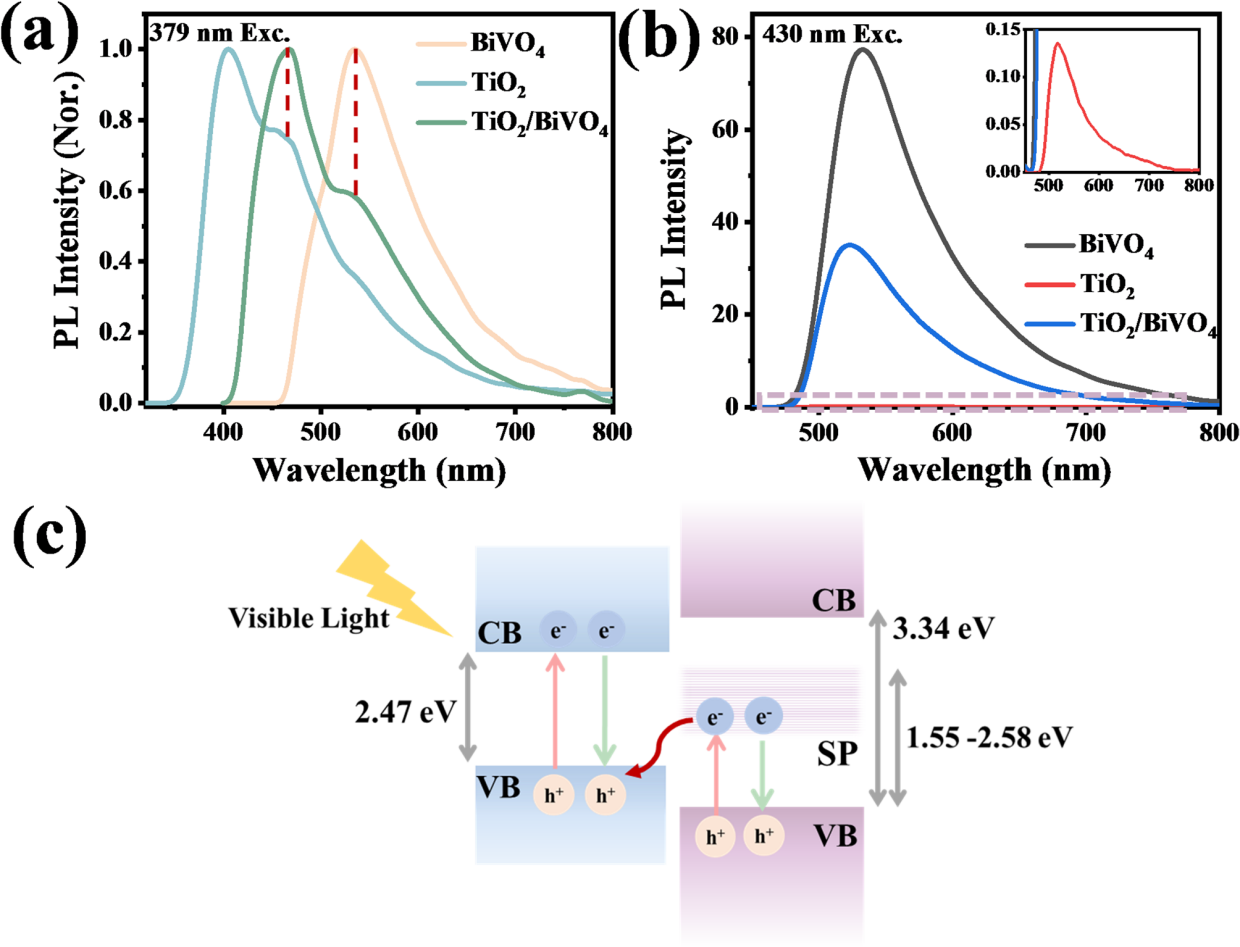
The presence of mid-gap states was further proved by PL spectra. (**a**) PL spectra of TiO_2_, BiVO_4_ and TiO_2_/BiVO_4_ under 379 nm excitation. (**b**) Comparison of PL spectra of the TiO_2_, BiVO_4_ and TiO_2_/BiVO_4_ with a 430 nm laser illumination. Inset: An enlarged view of the spectrum in the virtual box region. (**c**) Speculated charge transfer in TiO_2_/BiVO_4_ under visible light irradiation.

Excitation wavelength-dependent steady-state PL spectra were then measured to further prove the presence of mid-gap states and determine their approximate positions. As shown in Fig. 6a, TiO_2_/BiVO_4_ composites show a strong fluorescence emission at 467 nm and a shoulder peak centered at 526 nm under 379 nm laser excitation, which can be attributed to the carrier recombination of the VB and CB of TiO_2_ and BiVO_4_, respectively. However, there has been a noticeable change in the PL spectra of TiO_2_/BiVO_4_ when excited by photons with energies lower than the band gap of TiO_2_ (Fig. 6b), and only one peak centered at 522 nm appears. The origin of this fluorescence peak can be obtained by comparing it with the PL spectra of pure BiVO_4_ and TiO_2_ under the same conditions. As shown in Fig. 6b, the PL spectrum of pure BiVO_4_ is not significantly affected by changing the excitation light, and the center wavelength is still 532 nm. However, it’s worth noting that pure TiO_2_ exhibits a weak fluorescence centered at 517 nm, which can be well explained by the recombination of the excited SP states (inset of Fig. 6b). Therefore, it is reasonable to assume that this low-energy PL emission of TiO_2_/BiVO_4_ around 522 nm originates from the fluorescence superposition of BiVO_4_ and SP states, while the shift of the central wavelength and the attenuated intensity compared to BiVO_4_ reflect the interaction and charge transfer between TiO_2_/BiVO_4_. Moreover, the different dependence of excitation wavelength of TiO_2_ and BiVO_4_ indicates that mid-gap bands exist in TiO_2_ rather than BiVO_4_. Based on the spectral range of fluorescence emission of TiO_2_ (inset of Figs. 6b and 480 ~ 800 nm), it is estimated that the energy levels corresponding to the SP states are about 1.55 ~ 2.58 eV above the valence band of TiO_2_.

According to the above discussions, we refined the band structure of TiO_2_/BiVO_4_ and added the SP states in TiO_2_. As shown in Fig. 6c, the small-polaronic mid-gap states (represented by SP in Fig. 6c) and VB of TiO_2_, together with CB and VB of BiVO_4_, display a zigzagged band structure arrangement, which is more beneficial in promoting carrier transport and enhancing electron-hole separation at the interface of heterojunction. Upon visible light illumination, BiVO_4_ would be excited to generate electron-hole pairs, whereas electrons in TiO_2_ can be transformed from the VB into SP states. Since the potential of SP states of TiO_2_ (centered at ~ 0.5 eV vs. NHE) is more negative than the potential of VB of BiVO_4_ (1.86 eV vs. NHE), the accumulated photogenerated electrons at the SP states can smoothly recombine with the holes on the VB of BiVO_4_ through the S-scheme migration path driven by the internal electric field, band bending, and Coulombic attraction^30–32^. Consequently, the photogenerated electrons and holes are spatially separated and reserved on the CB of BiVO_4_ and VB of TiO_2_, respectively.

### Mid-gap states induced charge transfer in TiO_2_/BiVO_4_

**Fig. 7:**
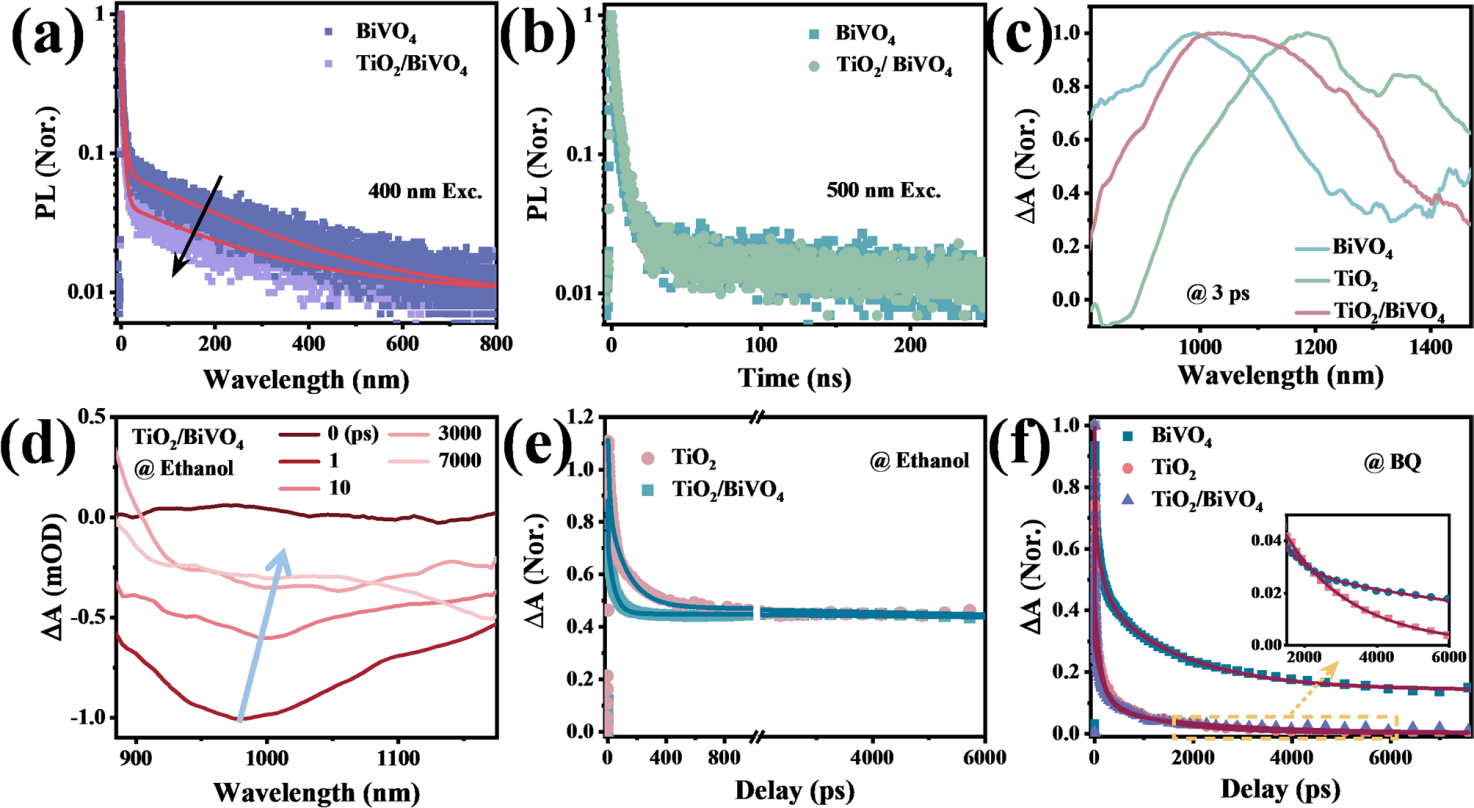
Detailed carrier dynamics in TiO_2_/BiVO_4_ heterostructures, and the presence of localized polaronic mid-gap states can enhance the interfacial carrier transfer and recombination in TiO_2_/BiVO_4_ heterojunctions. PL kinetics of BiVO_4_ in BiVO_4_ and TiO_2_/BiVO_4_ under (**a**) 400 nm and (**b**) 500 nm excitation. The solid curves represent the fitting results using the parameters listed in Table S1. (**c**) TA spectra of TiO_2_, BiVO_4_ and TiO_2_/BiVO_4_ collected at 3 ps. The TA spectra of TiO_2_/BiVO_4_ contain contributions from both TiO_2_ and BiVO_4_. (**d**) TA spectra of TiO_2_/BiVO_4_ in ethanol at indicated delay times under 430 nm excitation, exhibiting a red shift with the increase of delay time. (**e**) Normalized TA kinetics of TiO_2_ and TiO_2_/BiVO_4_ in ethanol probed at 952 nm, reflecting the decay of electrons. (**f**) Corresponding kinetics of holes probed at 1100 nm. Solid lines show the results of exponential fitting by using the parameters listed in the figure and Table S2.

To gain deep insight into the detailed carrier dynamics and photophysical process in TiO_2_/BiVO_4_ heterostructures, time-resolved PL and femtosecond transient absorption (fs-TA) measurements were carried out to investigate the photoinduced carrier dynamics of different systems. Figure 7a and b show the comparative study of the PL kinetics of BiVO_4_ in pure BiVO_4_ and TiO_2_/BiVO_4_ with excited (400 nm Exc.) and unexcited SP states (500 nm Exc.). It can be observed that the decay becomes faster in TiO_2_/BiVO_4_ at 400 nm excitation (Fig. 7a), and the average lifetime of BiVO_4_ is reduced from 17.3 ns to 8.9 ns after loading TiO_2_ (Table S1). The shortened degree of PL lifetime of BiVO_4_ in TiO_2_/BiVO_4_ (~ 51%) is close to the quenched fluorescence shown in Fig. 6b, indicating that the two changes come from the same dynamic process. Extraction and more efficient recombination of carriers can both lead to the reduced fluorescence intensity and lifetime. However, BiVO_4_ exhibits similar fluorescence decay kinetics in pure BiVO_4_ and TiO_2_/BiVO_4_ when the SP states are not excited (Fig. 7b), which excludes the possible electron or hole transfer from BiVO_4_ to TiO_2_ and confirms the necessity of excited SP states. Hence, we believe that the interfacial recombination of photogenerated electrons in SP states and holes in BiVO_4_ is the main reason for the differential PL lifetime and intensity, supporting the model we proposed.

Carrier dynamics at faster time scales were observed by carrying out femtosecond transient absorption (fs-TA) measurements using a pump pulsed laser with photon energy (430 nm, 2.88 eV) much smaller than the band gap of TiO_2_ (3.34 eV) and a continuum probe pulse in the near infrared (NIR) region (800 ~ 1500 nm)^33^. As shown in Fig. 7c, the fs-TA spectra of pristine BiVO_4_ and TiO_2_ are both dominated by a broad photoinduced absorption (PIA) signal centered at 990 nm and 1200 nm, respectively, while TiO_2_ also exhibits a weak negative signal centered at 835 nm. For TiO_2_/BiVO_4_ composites, the PIA wavelength range is broadened to the entire spectrum range and the central wavelength is shifted to 1050 nm. These TA features contain obvious contributions from both BiVO_4_ and TiO_2_, suggesting the existence of BiVO_4_ and TiO_2_ and confirming the successful formation of heterostructure. According to the steady state absorption spectra (Fig. S3), the broad PIA signal can be assigned to the excited-state absorption (ESA), and the negative bleach peak can be assigned to ground state bleach (GSB) caused by the state filling of carriers after excitation^34–36^.

We then distinguish the contribution of electrons and holes in the PIA and GSB signals by measuring transient absorption spectra in the presence of electron or hole scavengers in order to track the electron and hole behaviors separately. Following literature methods^28,37^we used ethanol as the hole scavenger and benzoquinone (BQ) as the electron scavenger. In the presence of hole scavenger (in ethanol), the TiO_2_/BiVO_4_ and pure TiO_2_ exhibit similar broad ground state bleach (GSB) (Fig. S4a), and no TA signal is observed for pure BiVO_4_, which indicates that the contributions of photogenerated electrons to the PIA signals are negligible, but are closely related to the GSB signals of TiO_2_/BiVO_4_ and TiO_2_. Notably, unlike pure TiO_2_, the GSB signal of TiO_2_/BiVO_4_ is redshifted with increasing delay time, and the peak position shifted from ∼980 to ∼1035 nm (Fig. 7d and S4b). This shift may be attributed to the bandgap modification of TiO_2_ as a result of the strong electronic coupling between the BiVO_4_ and the TiO_2_, that is, the fast electron transfer process from TiO_2_ to BiVO_4_ leads to an unbalanced carrier distribution in TiO_2_/BiVO_4_ and therefore the altered structure of transient absorption spectra^1,37,38^.

The rate of electron transfer can be obtained by fitting the TA kinetics probed at 952 nm. As shown in Fig. 7e, the GSB signals of TiO_2_/BiVO_4_ and TiO_2_ are both fitted by three-exponential functions using the parameters shown in the table inset the figure. The TA kinetics of TiO_2_/BiVO_4_ and TiO_2_ show two similar time constants of ~ 0.64 ps and > 10 ns, which can be corresponded to the intrinsic electron trapping and recombination processes in the TiO_2_ component, respectively. In addition, TiO_2_/BiVO_4_ also yields a faster time constant of τ_2_ (~ 45 ps) than pure TiO_2_ (τ_2_ ~ 101 ps), indicating a new decay pathway for electrons in the TiO_2_/BiVO_4_ heterojunction, i.e., the interfacial electron transfer process^20^. The ET rate is approximately estimated to be ~ 0.01 ps^− 1^ in terms of Eq. (2).

2 $$k_{{ET}} = {\text{ 1}}/\tau _{{\text{2}}} ^{'} - {\text{1}}/\tau _{{\text{2}}}$$

where τ_2_^’^ and τ_2_ are the τ_2_ time constants of TiO_2_ in pure TiO_2_ and TiO_2_/BiVO_4_, respectively. In addition, we do not observe the electron transfer process from BiVO_4_ to TiO_2_ in TiO_2_/BiVO_4_, as there is no slower rising edge in TiO_2_/BiVO_4_ (Fig. S4c).

The dynamic processes associated with photogenerated holes are also stripped out by the TA results measured with the electron scavenger BQ. BiVO_4_, TiO_2_ and TiO_2_/BiVO_4_ crystals all exhibit broad positive spectra that decay with increasing delay time, with central wavelengths around 1080 nm, 1300 nm and 1240 nm, respectively (Fig. S5), corresponding to the PIA of holes. In Fig. 7f, the kinetics of TiO_2_/BiVO_4_, BiVO_4_ and TiO_2_ under the same irradiation condition are compared and fitted by triexponential functions with the time constants listed in Table S2. The lifetime of photogenerated holes in TiO_2_/BiVO_4_ crystals is measured to be ~ 196 ps, which is much shorter than the lifetime of ~ 735 ps in pure BiVO_4_ and slightly longer than the lifetime of ~ 156 ps in pure TiO_2_. More significantly, the long-lived component τ_3_ is extended to ~ 2187 ps after the formation of the heterojunction. These results rule out the possibility of hole transfer from TiO_2_ to TiO_2_/BiVO_4_ and indicate that, in TiO_2_/BiVO_4_, the decay of photogenerated holes in BiVO_4_ is accelerated, accompanied by the generation of long-lived photogenerated holes in TiO_2_, which is well combined with our expectations. That is, there is an ET from TiO_2_ to BiVO_4_ and photogenerated electrons on the SP states of TiO_2_ can recombine with the holes on the VB of BiVO_4_, resulting in the long-lived separated holes left in TiO_2_.

### Photocatalytic mechanism over TiO_2_/BiVO_4_

**Fig. 8:**
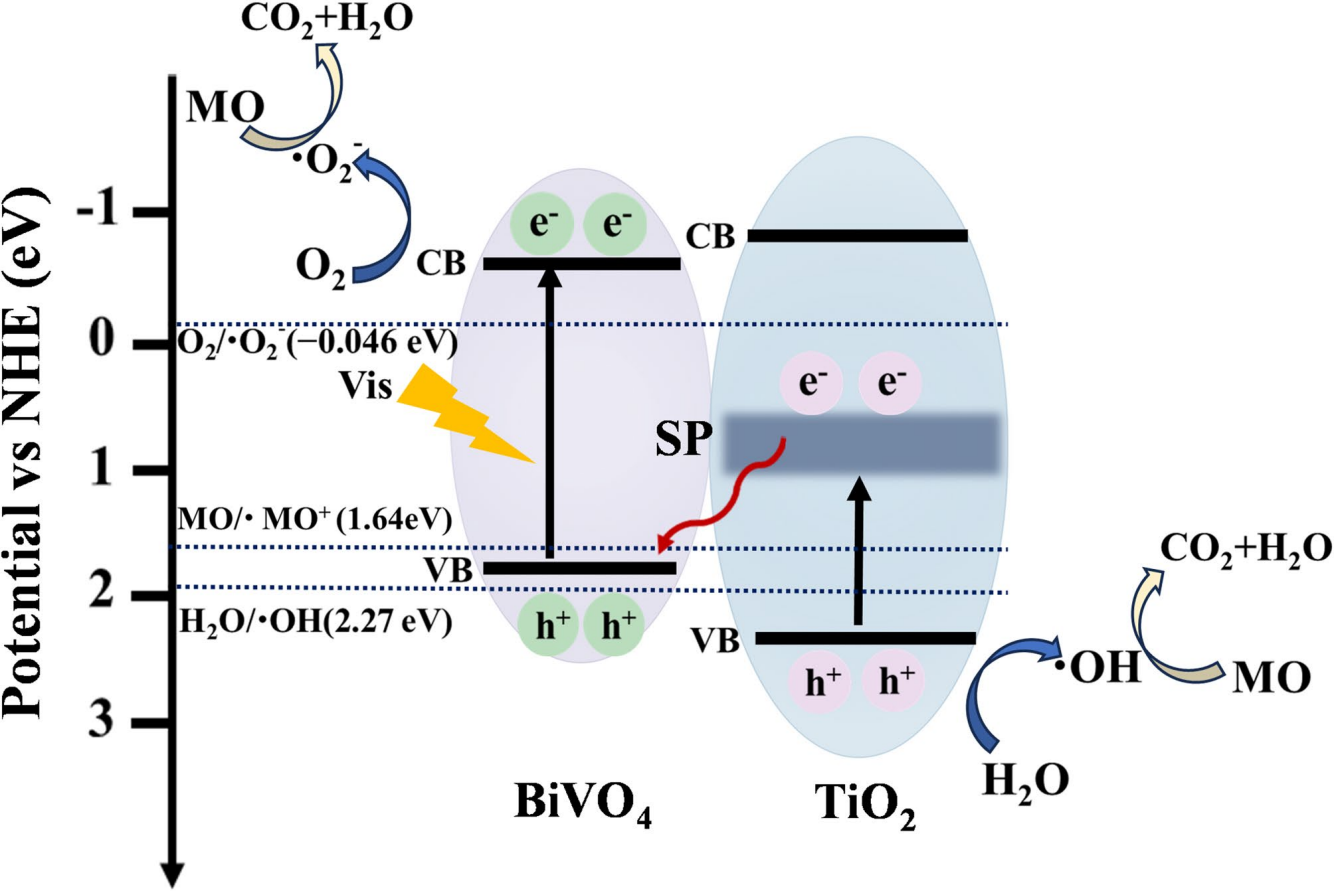
Proposed photocatalytic reaction processes and charge transfer of TiO_2_/BiVO_4_. The separation and transfer of the photogenerated electron-hole pairs followed a direct S-scheme path induced by localized polaronic mid-gap states. The efficient elimination of electrons in TiO_2_ and holes in BiVO_4_ can prolong the lifetimes of the remaining carriers and lead to the enhancement of photocatalytic activity of TiO_2_/BiVO_4_.

The above experimental results unambiguously confirm the model presented in Fig. 6c and the role of small-polaronic mid-gap states in the enhancement of photocatalytic performance of TiO_2_/BiVO_4_. The possible mechanism of MO degradation can be concluded as shown in Fig. 8. Due to the existence of SP states, both TiO_2_ and BiVO_4_ can be photoexcited to generate electron–hole pairs for the fabricated TiO_2_/BiVO_4_ composites. The separation and transfer of the photoinduced electron-hole pairs follows a direct S-scheme path, in which the accumulated electrons on the SP states of TiO_2_ would thermodynamically transfer to the VB of BiVO_4_ and recombine with photoexcited holes^13,17^. Consequently, the photogenerated electrons on the CB of BiVO_4_ and holes on the VB of TiO_2_ are spatially separated and reserved. These preserved carriers with strong redox ability can be used to produce active species with strong oxidation ability or oxidize MO directly, which eventually leads to the significant improvement of the performance of TiO_2_/BiVO_4_ for photocatalytic degradation of MO.

In the photocatalytic degradation reaction of methyl orange (MO), the electrons on the CB of BiVO_4_ (− 0.61 eV vs. NHE) have adequate potential to react with O_2_ to generate superoxide anion radicals (·O_2_^–^) (− 0.046 eV vs. NHE), and hydroxyl radicals (OH) can be further formed due to the reaction of water molecules (2.27 eV vs. NHE)^39–41^. Besides, holes left on the VB of TiO_2_ (2.65 eV vs. NHE) exhibit strong oxidation ability and would either oxidize MO molecules (1.64 eV vs. NHE)^42^ or react with water molecules to engender ·OH. Finally, the MO molecules are eventually decomposed into CO_2_ and H_2_O under the action of active substances, namely holes, superoxide anion radicals and hydroxyl radicals.

## Conclusions

In summary, the TiO_2_/BiVO_4_ nanocomposites with large energy barrier developed in this work demonstrate exceptional visible-light photocatalytic performance, achieving ~ 100% methyl orange degradation rate within 1 h under visible light. Unique charge transfer in Type-I TiO_2_/BiVO_4_ heterojunction enabled by polarons was confirmed by comprehensive characterizations. Time-resolved spectra revealed the critical role of SP states in facilitating ultrafast electron transfer (k_ET_ ~ 0.01 ps^− 1^) from TiO_2_ to BiVO_4_ and promoting charge separation, which effectively prolonged the lifetimes of photogenerated carriers and eventually led to the outstanding visible photocatalytic activity of TiO_2_/BiVO_4_. These findings fundamentally advance our understanding of polaron-mediated photocatalysis by establishing how strategically introduced mid-gap states can simultaneously optimize visible-light harvesting and charge separation in heterojunction systems. The mechanistic insights and material design principles presented here provide a robust foundation for developing high-efficiency photocatalysts for environmental and energy applications.

## Electronic supplementary material

Below is the link to the electronic supplementary material.

Supplementary Material 1

## Data Availability

The datasets used and/or analyzed during the current study are available from the corresponding author on reasonable request.

## References

[1] Zhang, C. et al. Charge separation by creating band bending in metal–organic frameworks for improved photocatalytic hydrogen evolution. *Angew Chem. Int. Ed.* **61**, e202204108 (2022). 10.1002/anie.202204108 35522460

[2] Bariki, R., Pradhan, S. K., Panda, S., Nayak, S. K. & Pati, A. R. Hierarchical UiO-66(– NH_2_)/CuInS_2_ S-Scheme photocatalyst with controlled topology for enhanced photocatalytic N_2_ fixation and H_2_O_2_ production. *Langmuir* **39**, 7707–7722 (2023). 37212348 10.1021/acs.langmuir.3c00519

[3] Yuan, L., Qi, M. Y., Tang, Z. R. & Xu, Y. J. Coupling strategy for CO_2_ valorization integrated with organic synthesis by heterogeneous photocatalysis. *Angew Chem. Int. Ed.* **60**, 21150–21172 (2021). 10.1002/anie.202101667 33908154

[4] Xu, Q., Zhang, L., Cheng, B., Fan, J. & Yu, J. S-Scheme heterojunction photocatalyst. *Chem* **6**, 1543–1559 (2020).

[5] Pihosh, Y. et al. Photocatalytic generation of hydrogen by core-shell WO_3_/BiVO_4_ nanorods with ultimate water splitting efficiency. *Sci. Rep.* **5**, 11141 (2015). 26053164 10.1038/srep11141 PMC4459147

[6] Banyal, R. et al. Synergetic photocatalytic degradation of the Tetracycline antibiotic over S-scheme based BiOBr/CuInS_2_/WO_3_ ternary heterojunction photocatalyst. *Solid State Sci.* **157**, 107700 (2024).

[7] Hasija, V. et al. Dual S-scheme Bi_2_MoO_6_/g-C_3_N_4_/Ag_2_MoO_4_ ternary heterojunction: interfacial charge transfer, broadband spectrum, enhanced redox ability. *Solid State Sci.* **157**, 107693 (2024).

[8] Wu, C. et al. Mechanistic study of B-TiO_2_/BiVO_4_ S-scheme heterojunction photocatalyst for Tetracycline hydrochloride removal and H_2_ production. *Sep. Purif. Technol.* **312**, 123398 (2023).

[9] Das, D. & Shyam, S. Reduced work function in anatase ⟨101⟩ TiO_2_ films Self-Doped by O-Vacancy-Dependent Ti^3+^ bonds controlling the photocatalytic dye degradation performance. *Langmuir* **40**, 10502–10517 (2024). 38711250 10.1021/acs.langmuir.4c00028

[10] Kwon, J. et al. Improved charge carrier dynamics by unconventional doping strategy for BiVO_4_ photoanode. *Small Sci* 2500051 (2025). 10.1002/smsc.202500051 PMC12257881 40666032

[11] Hanif, M. B. et al. 2D TiO_2_ nanosheets decorated via sphere-like BiVO_4_: A promising non-toxic material for liquid phase photocatalysis and bacterial eradication. *ChemSusChem* **17**, e202400027 (2024). 38588020 10.1002/cssc.202400027

[12] Liaqat, M. et al. Synergistic photocatalytic activity of TiO_2_/BiVO_4_ nanocomposites: optimization, characterization, and recyclability for dye and antibiotic degradation. *J. Inorg. Organomet. Polym.* **34**, 3246–3257 (2024).

[13] Wei, X. P., Yang, Y. T., Zheng, Z. Y., Yuan, W. B. & Ni, H. G. A simple Preparation method of Ti/TiO_2_/BiVO_4_ and implications for enhanced photoelectrocatalytic performance under visible light illumination. *Inorg. Chem. Commun.* **171**, 113602 (2025).

[14] Chen, R. et al. Enhanced photocatalytic activity of oxygen vacancy modulation interfacial electric field in S-scheme heterojunction VO/BiVO_4_-TiO_2_ and its mechanism. *Appl. Surf. Sci.* **665**, 160322 (2024).

[15] Rana, A. et al. Integrating BiOI/g-C_3_N_4_/Bi_2_WO_6_ derived dual S-Scheme photocatalyst with Biochar for emerging adsorption for photocatalysis: multicharge migration and mechanistic insights. *Ind. Eng. Chem. Res.* **63**, 6960–6973 (2024).

[16] Rana, A. et al. Novel S-scheme derived Mo–Bi_2_WO_6_/WO_3_/Biochar composite for photocatalytic removal of methylene blue dye. *J. Phys. Chem. Solids*. **196**, 112385 (2025).

[17] Xie, M. et al. Long-lived, visible-light-excited charge carriers of TiO_2_/BiVO_4_ nanocomposites and their unexpected photoactivity for water splitting. *Adv. Energy Mater.* **4**, 1300995 (2013).

[18] Hu, Y. et al. BiVO_4_/TiO_2_ nanocrystalline heterostructure: A wide spectrum responsive photocatalyst towards the highly efficient decomposition of gaseous benzene. *Appl. Catal. B: Environ.* **104**, 30–36 (2011).

[19] Drisya, K. T. et al. Electronic and optical competence of TiO_2_/BiVO_4_ nanocomposites in the photocatalytic processes. *Sci. Rep.* **10**, 13507 (2020). 32782289 10.1038/s41598-020-69032-9 PMC7421441

[20] Lettieri, S., Pavone, M., Fioravanti, A., Amato, L. S. & Maddalena, P. Charge carrier processes and optical properties in TiO_2_ and TiO_2_-based heterojunction photocatalysts: A review. *Materials* **14**, 1645 (2021). 33801646 10.3390/ma14071645 PMC8036967

[21] Hruska, E., Husek, J., Bandaranayake, S. & Baker, L. R. Visible light absorption and hot carrier trapping in anatase TiO_2_: the role of surface oxygen vacancies. *J. Phys. Chem. C*. **126**, 10752–10761 (2022).

[22] Tamaki, Y. et al. Dynamics of efficient electron–hole separation in TiO_2_ nanoparticles revealed by femtosecond transient absorption spectroscopy under the weak-excitation condition. *Phys. Chem. Chem. Phys.* **9**, 1453–1460 (2007). 17356752 10.1039/b617552j

[23] Morgan, B. J., Scanlon, D. O. & Watson, G. W. Small polarons in Nb- and Ta-doped rutile and anatase TiO_2_. *J. Mater. Chem.* **19**, 5175–5178 (2009).

[24] Di Valentin, C. & Selloni, A. Bulk and surface polarons in photoexcited anatase TiO_2_. *J. Phys. Chem. Lett.* **2**, 2223–2228 (2011).

[25] Wang, S. et al. New BiVO_4_ dual photoanodes with enriched oxygen vacancies for efficient solar-driven water splitting. *Adv. Mater.* **30**, e1800486 (2018). 29602201 10.1002/adma.201800486

[26] Liu, P. P. et al. TiO_2_–BiVO_4_ heterostructure to enhance photoelectrochemical efficiency for sensitive aptasensing. *ACS Appl. Mater. Interfaces*. **9**, 27185–27192 (2017). 28759199 10.1021/acsami.7b07047

[27] Yaghoubi, H. et al. Toward a visible light-driven photocatalyst: the effect of midgap-states-induced energy gap of undoped TiO_2_ nanoparticles. *ACS Catal.* **5**, 327–335 (2014).

[28] Kafizas, A. et al. Where do photogenerated holes go in anatase:rutile TiO_2_? A transient absorption spectroscopy study of charge transfer and lifetime. *J. Phys. Chem. A*. **120**, 715–723 (2016). 26777898 10.1021/acs.jpca.5b11567

[29] Song, X. et al. The Midas touch transformation of TiO_2_ nanowire arrays during visible light photoelectrochemical performance by carbon/nitrogen coimplantation. *Adv. Energy Mater.* **8**, 1800165 (2018).

[30] Cheng, C. et al. An inorganic/organic S-scheme heterojunction H_2_-production photocatalyst and its charge transfer mechanism. *Adv. Mater.* **33**, 2100317 (2021). 10.1002/adma.202100317 33904199

[31] Yang, Y., Cheng, B., Yu, J., Wang, L. & Ho, W. TiO_2_/In_2_S_3_ S-scheme photocatalyst with enhanced H_2_O_2_-production activity. *Nano Res.* **16**, 4506–4514 (2021).

[32] Wu, X., Chen, G., Wang, J., Li, J. & Wang, G. Review on S-scheme heterojunctions for photocatalytic hydrogen evolution. *Acta Phys. -Chim Sin*. **39**, 2212016 (2023).

[33] Miao, T. J. & Tang, J. Characterization of charge carrier behavior in photocatalysis using transient absorption spectroscopy. *J. Chem. Phys.* **152**, 194201 (2020). 33687236 10.1063/5.0008537

[34] Liu, H., Liu, M., Nakamura, R. & Tachibana, Y. Primary photocatalytic water reduction and oxidation at an anatase TiO_2_ and Pt-TiO_2_ nanocrystalline electrode revealed by quantitative transient absorption studies. *Appl. Catal. B: Environ.* **296**, 120226 (2021).

[35] Cooper, J. K., Reyes-Lillo, S. E., Hess, L. H., Jiang, C. M. & Neaton, J. B. Sharp, I. D. Physical origins of the transient absorption spectra and dynamics in thin-film semiconductors: the case of BiVO_4_. *J. Phys. Chem. C*. **122**, 20642–20652 (2018).

[36] Katoh, R., Murai, M. & Furube, A. Transient absorption spectra of nanocrystalline TiO_2_ films at high excitation density. *Chem. Phys. Lett.* **500**, 309–312 (2010).

[37] Yu, L. et al. The degradation mechanism of Methyl orange under photo-catalysis of TiO_2_. *Phys. Chem. Chem. Phys.* **14**, 3589–3595 (2012). 22310904 10.1039/c2cp23226j

[38] Xu, F. et al. Unique S-scheme heterojunctions in self-assembled TiO_2_/CsPbBr_3_ hybrids for CO_2_ photoreduction. *Nat. Commun.* **11**, 4613 (2020). 32929077 10.1038/s41467-020-18350-7 PMC7490390

[39] Liu, S., Bu, Y., Cheng, S., Tao, Y. & Hong, W. Preparation of g-C_3_N_5_/g-C_3_N_4_ heterojunction for Methyl orange photocatalytic degradation: mechanism analysis. *J. Water Process. Eng.* **54**, 104019 (2023).

[40] Yan, Z. et al. Interpreting the enhanced photoactivities of 0D/1D heterojunctions of cds quantum dots/TiO_2_ nanotube arrays using femtosecond transient absorption spectroscopy. *Appl. Catal. B: Environ.* **275**, 119151 (2020).

[41] Shi, H. et al. Construction of bi/polyoxometalate doped TiO_2_ composite with efficient visible-light photocatalytic performance: mechanism insight, degradation pathway and toxicity evaluation. *Appl. Surf. Sci.* **615**, 156310 (2023).

[42] Trandafilovic´, D. J. J. L. V. et al. Enhanced photocatalytic degradation of Methylene blue and Methyl orange by zno:eu nanoparticles. *Appl. Catal. B: Environ.* **00**, 1–26 (2016).

