# Supplementary material for: Unveiling the role of localized polaronic mid-gap states in enhanced carrier transfer in TiO2/BiVO4 heterojunctions under visible light irradiation
Source: Sci Rep. 2025 Jul 8;15:24343. doi: 10.1038/s41598-025-10259-9 (PMC12234835; doi:10.1038/s41598-025-10259-9)
Supplement: Supplementary file 1 — Supplementary Material 1 [file 41598_2025_10259_MOESM1_ESM.docx]

Supporting Information for

**Unveiling the Role of Localized Polaronic Mid-Gap States in Enhanced Carrier Transfer in TiO_2_/BiVO_4_ Heterojunctions under Visible Light Irradiation**

Zixi Yin^1, 2, 3, 5, *^ Xingchen Liu^1,2, 3, 5^, Guijie Liang^1, 2^^, 3^ Yin Wang^4^

^1^Hubei Key Laboratory of Low Dimensional Optoelectronic Materials and Devices, Hubei University of Arts and Science, Xiangyang 441053, Hubei, China

^2^Institute of functional materials, Hubei University of Arts and Science, Xiangyang 441053, Hubei, China

^3^Hubei Longzhong Laboratory, Xiangyang, 441053, Hubei, China

^4^School of Physics and Electronic Engineering, Hubei University of Arts and Science, Xiangyang 441053, Hubei, China

^*^Email: [yinzixi@hbuas.edu.cn](mailto:yinzixi@hbuas.edu.cn)

^5^These authors contributed equally to this work.

Fig. S1. SEM images of (a) TiO_2_ and (b) BiVO_4_.

Fig. S2. (a) Plot of (αhv)^2^ versus photon energy (h*v*) and (b) the Mott-Schottky plots of TiO_2_ and BiVO_4_.

Fig. S3. Diffuse reflection spectra of pure TiO_2_, BiVO_4_ and TiO_2_/BiVO_4_ in the near infrared (NIR) region.

Fig. S4. (a) Compare of TA spectra of TiO_2_/BiVO_4_ and TiO_2_ in ethanol at 1 ps. (b) TA spectra of TiO_2_ in ethanol at indicated delay times under 430 nm excitation. (c) TA decays of holes in TiO_2_ and TiO_2_/BiVO_4_ observed in ethanol within the first 10 ps, and the rise time of TiO_2_/BiVO_4_ has not slowed down significantly.

Fig. S5. (a) Comparison of TA spectra of holes in TiO_2_, BiVO_4_ and TiO_2_/BiVO_4_ observed in the presence of BQ after 430 nm excitation. TA spectra of (b) TiO_2_/BiVO_4_, (c) TiO_2_ and (d) BiVO_4_ in the presence of BQ at indicated delay times under 430 nm excitation.

| Sample (@534 nm) | a_1_ | τ_1_ (ns) | a_2_ | τ_2_ (ns) | a_3_ | τ_3_ (ns) | τ_Ave._ (ns) |
| --- | --- | --- | --- | --- | --- | --- | --- |
| BiVO_4_ | **0.65** | **1.34** | **0.29** | **6.26** | **0.06** | **244** | **17.3** |
| TiO_2_/BiVO_4_ | **0.69** | **0.95** | **0.28** | **5.12** | **0.03** | **227** | **8.90** |

**Table S1.** Multiexponential fitting parameters for the PL kinetics of BiVO_4_ in BiVO_4_ and TiO_2_/BiVO_4_ collected at 534 nm shown in Fig. 7a, where τ_n_ and a_n_ (n = 1, 2, 3) are the fitting lifetimes (ns) and their corresponding amplitudes; The τ_Ave._ is the average lifetime.

| Sample (@ 1100 nm) | a_1_ | τ_1_ (ps) | a_2_ | τ_2_ (ps) | a_3_ | τ_3_ (ps) | τ_Ave._ (ps) |
| --- | --- | --- | --- | --- | --- | --- | --- |
| TiO_2_ | **0.63** | **1.2** | **0.27** | **121** | **0.10** | **1221** | **156** |
| BiVO_4_ | **0.31** | **17** | **0.29** | **167** | **0.40** | **1702** | **735** |
| TiO_2_@BiVO_4_ | **0.72** | **1.02** | **0.21** | **201** | **0.07** | **2187** | **196** |

**Table S2.** Multiexponential fitting parameters for the kinetics of holes in TiO_2_, BiVO_4_ and TiO_2_/BiVO_4_ probed at 1100 nm shown in Figure 7f, where τ_n_ and a_n_ (n = 1, 2, 3) are the fitting lifetimes (ps) and their corresponding amplitudes; The τ_Ave._ is the average lifetime.
